# Looking into the world’s largest elephant population in search of ligninolytic microorganisms for biorefineries: a mini-review

**DOI:** 10.1186/s13068-022-02159-1

**Published:** 2022-06-10

**Authors:** Bame Rammala, Nerve Zhou

**Affiliations:** Department of Biological Sciences and Biotechnology, Botswana International University of Science and Technology, Private Bag 16, Palapye, Botswana

**Keywords:** Lignin, Lignocellulosic biomass, Biorefinery, Depolymerisation, Ligninolytic microorganisms, Elephant gastrointestinal tract

## Abstract

Gastrointestinal tracts (GIT) of herbivores are lignin-rich environments with the potential to find ligninolytic microorganisms. The occurrence of the microorganisms in herbivore GIT is a well-documented mutualistic relationship where the former benefits from the provision of nutrients and the latter benefits from the microorganism-assisted digestion of their recalcitrant lignin diets. Elephants are one of the largest herbivores that rely on the microbial anaerobic fermentation of their bulky recalcitrant low-quality forage lignocellulosic diet given their inability to break down major components of plant cells. Tapping the potential of these mutualistic associations in the biggest population of elephants in the whole world found in Botswana is attractive in the valorisation of the bulky recalcitrant lignin waste stream generated from the pulp and paper, biofuel, and agro-industries. Despite the massive potential as a feedstock for industrial fermentations, few microorganisms have been commercialised. This review focuses on the potential of microbiota from the gastrointestinal tract and excreta of the worlds’ largest population of elephants of Botswana as a potential source of extremophilic ligninolytic microorganisms. The review further discusses the recalcitrance of lignin, achievements, limitations, and challenges with its biological depolymerisation. Methods of isolation of microorganisms from elephant dung and their improvement as industrial strains are further highlighted.

## Background

With over 130,000 elephants, Botswana has over a third of Africa’s population of elephants and the largest in the whole world [1]. Negative impacts of big herds of elephants such as environmental damage through destruction of vegetation by trampling and foraging [2] are major cause of concern. Elephants forage for more than 18 h and consume between 100 and 300 kg of vegetation in a day. This foraging strategy involves the consumption of large quantities of poor-quality forage to sustain their huge metabolic requirements [3–5]. For efficient fermentative digestion, elephants harbour microorganisms, which compensate for their inability to break down the recalcitrant ligninolytic plant diet [5]. There is potential in tapping such symbiotic associations involving the gut microbiota of the largest population of elephants to solve the environmental challenge associated with bulky lignin waste streams generated from the pulp and paper, biofuel- and agro-industries. Although other ways of utilising lignin wastes are well documented [6], the use of lignin as a carbon substrate for microbial fermentation in a biorefinery valorisation strategy is sustainably attractive. The recalcitrance of lignin to microbial breakdown has remained as the major drawback. Although other strategies to develop ligninolytic microorganisms are possible, the search for efficient and industrially suitable ligninolytic microorganisms from natural environments is attractive [7–10]. Literature suggests that the gut microbiota of animals with a lignocellulosic diet is very promising in the search for potential ligninolytic microorganisms [11–13]. The isolation of microorganisms from gastrointestinal tracts of animals with preferential carbon utilisation ranges for the development of industrial applications is well a documented strategy [14–17]. This review uniquely focuses on the potential of isolation of robust ligninolytic microorganisms from the world´s largest population of elephants in Botswana. The wider choice of elephants to isolate potential ligninolytic microorganisms from increases the chance to find novel degradation pathways. Botswana has an arid to semi-arid climate, resulting in desert-like conditions for about a third of the country and highly variable rainfall patterns [18]. The extreme environmental conditions in Botswana provide a selective pressure for extremophilic microorganisms, which could minimise the costs of pre-treatment processes of lignin depolymerisation and improve the efficiency of the production process where stress-tolerant and robust commercial-scale lignin degradation processes are required. In addition to presenting elephants as a potential source of ligninolytic microorganisms, this review further addresses the gaps and challenges in the biorefinery approach to valorise waste lignin by highlighting the current sources of ligninolytic microbes of animal guts origin and describing the development of ligninolytic isolates from elephant guts as well as advances in isolation of ligninolytic microorganisms from herbivore dung. The review further discusses the potential of elephant gut-associated symbionts as extremophilic industrial-scale production strains, as elephants of Botswana inhabit extremely hot and semi-arid to arid environments and migrate hundreds of kilometres around the region further subjecting the symbionts to a wide range of selection pressures. The extremophilic traits as key traits for desirable strains for an efficient and sustainable biorefinery are further discussed. Lastly, the review gives an overview of approaches that can be used to improve the ligninolytic properties of elephant gut-associated strains.

## Lignin waste stream background

The valorisation of abundant lignin from agro and industrial processing, considered as wastes, has been a topic for research. About 130 million tonnes of lignin are produced from the pulp and paper industry annually [8, 19, 20]. In addition, the second generation of bioethanol from lignocellulosic feedstocks may generates about 70,000 tonnes of lignin annually [21]. Furthermore, lignin, one of the major components of the lignocellulosic biomass (LCBM) [22], is the second most abundant source of carbon on earth after cellulose [23–22]. With climate change and the need to reduce dependence on petroleum fuels, lignin waste generation during bioethanol production is projected to increase rapidly to approximately 225 million tonnes by 2030 [26]. About 1 kg lignin is generated as a byproduct from every liter of bioethanol produced [27]. Waste lignin routes of utilisation either are not environmentally friendly or cannot cope with the abundant amounts being produced. Some industries discard lignin as wastewater to the detriment of marine life as it accumulates in water bodies [28, 29]. Valorisation of this “waste” stream remains limited in market and product diversity. The most predominant methods of lignin upgrading in a biorefinery concept are either thermochemical: pyrolysis, hydrogenolysis, hydrothermal liquefaction, physical: steam explosion, mechanical grinding, combustion [6, 30] or chemical: acid, base or metallic catalysts, oxidative lignin depolymerisation [31]. Although these methods produce a wide range of products of importance such as pyro char, bio-oil, ethylene benzene and others [6], they have several drawbacks such as high-energy demands, complicated process control and complex product formation due to the general inertness of lignin [6, 32]. Moreover, the poorly established methods may lead to other modifications to lignin resulting in increased recalcitrance and subsequent difficulty in the separation of the desired products from the repolymerised lignin [21, 33, 34, 35]. In a world of a fast-growing population, dwindling resources and climate change, such unsustainable methods of lignin depolymerisation are being challenged by a much more attractive biological valorisation approach. Research increasingly suggests that some microorganisms have natural ligninolytic capabilities [36, 37, 38, 39, 40]. Using microorganisms as cell factories to depolymerise lignin in a biorefinery concept [41, 42, 43, 44, 45, 46] generates negligible wastes (environmentally friendly), reduces greenhouse gas emissions, and produces several speciality chemicals (from many metabolic pathways) which feeds into an attractive circular bioeconomy [24, 27, 47]. The gastrointestinal tract provides an anaerobic environment, which further influences the possibility of finding robust anaerobic ligninolytic microorganisms. However, anaerobic lignin depolymerisation is still unclear [48]. Bacteria able to degrade lignin under anaerobic conditions remain a minority [49], but a novel facultative bacterium, *Tolumonas lignolytica* has been investigated for lignin depolymerisation under anaerobic conditions [50], although the strain was isolated from tropical rain forest soil [51].

## Lignin depolymerisation using ligninolytic microorganisms: limitations and challenges

**Fig. 1:**
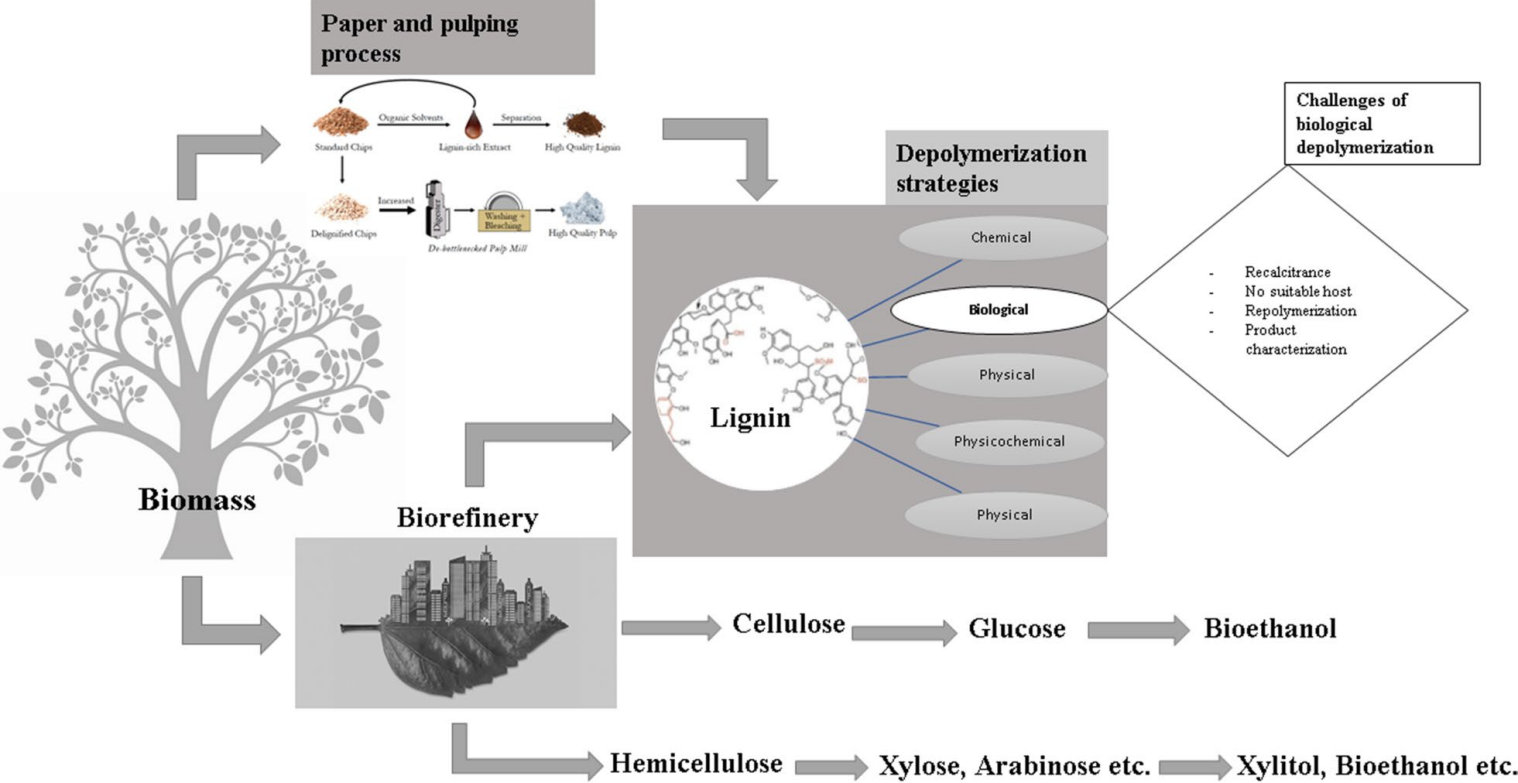
Highlights lignocellulosic biomass pretreatment leading to the release of cellulose, hemicellulose (their value-added products) and lignin. Some of the challenges of biological lignin valorisation are highlighted. Adapted and modified from [52, 53]

The major bottleneck to the microbial valorisation of lignin as outlined in Fig. 1, is the challenge to find robust lignin-degrading microorganisms despite the attractiveness of biological depolymerisation.

The recalcitrance of lignin to microbial breakdown is one major challenge hindering the utilisation of the inexpensive waste stream [54]. Literature on lignin generated from different industries suggests that about only a meagre 2% of the lignin substrate is turned into various value-added products such as adhesives, additives, vanillin, carbon fibre, polyurethane [33, 55, 56]. A consortium of microorganisms isolated from lignin-rich sources has been suggested to have the capability to overcome the recalcitrance challenge [57, 58]. A synergistic enzymatic and microbial lignin conversion alternative strategy to reduce the inefficiency of the process can be used [24]. Fungi such as *Phanerochaete chrysosporium*, *Aspergillus* sp. and *Trametes trogii* S0301 are naturally more efficient lignin depolymerisers than any other microorganisms that have been extensively studied [23, 59, 60, 61]. However, there are many challenges with the use of fungi for commercial-scale depolymerisation of lignin such as their requirement for co-substrates for mycelial growth, making the process more costly and therefore less scalable [62, 63]. Furthermore, fungi have a relatively slow growth rate and poorly adapt to industrial conditions (temperature, pH, and poor oxygen conditions) and are therefore difficult to cultivate [23]. In addition, the practical challenges of complex fungal protein expression and fungal genetic manipulation further point to the absence or lack thereof of commercialised fungal biocatalytic processes [32, 64]. Apart from fungi, bacteria genera such as *Streptomyces*, *Nocardia*, *Sphingobium*, *Rhodococcus, Pseudomonas, Serratia*, to name but a few, have been reported to have lignin-depolymerising capabilities [8] (Table 1) despite the poor availability of information and characterisation studies [65]. Compared to fungi, bacteria seldomly require co-substrates for growth, tolerate wider industrial parameters (pH, temperature, and absence of oxygen), have a short incubation period, have versatile ligninolytic pathways, are often amenable to genetic modifications, and adapt to changes in industrial conditions with relative ease than fungi [58, 66, 67, 68]. However, a notable gap when depolymerising lignin with bacteria is the limited performance as compared to fungi [31, 69, 70]. Typical examples of bacteria currently being used at the industry level are *Ralstonia eutropha* and *Pseudomonas putida* which have been used to produce PHAs at a large scale [71]. Apart from fungi and bacteria, yeasts such as *Rhodotorula glutinis*, *Rhodotorula vanillica*, *Trichosporon cutaneum* [71, 72] have been documented to utilise synthetic lignin and some lignin monomers to produce lipids. Yeasts have shown increased potential in valorising lignin-like dyes, have a higher growth rate than other microorganisms, a high endotoxin-free cell biomass and short processing time [73, 74]. However, yeasts do not natively secrete any haeme peroxidases [75, 76]. Recently, the possibility of engineered budding yeast *Saccharomyces cerevisiae* to produce coumarin from lignin was explored [77]. Of all the documented species able to degrade lignin, few have been commercialised or applied at the industrial level, [27]. Most lignin-degrading microorganisms grow under aerobic conditions [37]. Aerobic industrial fermentations, however, are less efficient because of the rate-limiting oxygen limitations caused by the low solubility of oxygen in water and other fermenting liquids [78]. The concentration of oxygen in large-scale bioreactors is kept adequate by increasing the agitation speed of impellers and aeration rate. The mechanical agitation processes are therefore energetically expensive, not compatible with use of filamentous fungal production strains and shear sensitive strains. Therefore, there is need for development of anaerobic valorisation of lignin to increase the productivity of the processes.

**Table 1:** Examples of bacteria documented to have lignin-degrading capabilities

Microorganism	Product(s)	References
*Acinetobacter sp.*	Alkanes, wax esters	[32, 79, 80]
*Bacillus sp.*	Mixed aromatic monomers	[10, 25, 60, 81]
*Citrobacter freundii*	Phenols	[82]
*Leucobacter sp.*		[80, 83]
*Nocardia sp.*	Ferulic acid, vanillic acid	[84, 85]
*Norosphingobium sp. B-7*	Vanillic acid, p-hydroxy benzoic acid	[41, 86]
*Propionibacterium sp.*		[36]
*Pseudomonas sp.*	Polyhydroxyalkanoates	[80, 87, 88]
*Rhodococcus jostii*	Chloro-benzoate, vanillin	[32, 60, 89, 90, 154]
*Serratia sp.*	Propanoic acid, 2-methyl-2,3-dihydro-1-H-benz [g] indole	[66, 91, 92]
*Stenotrophomonas sp.*		[93, 94]
*Streptomyces viridosporous*	Acid-precipitable polyphenolic polymeric lignin	[83, 95, 96]

## Elephant gastrointestinal tract as possible source of extremophilic ligninolytic microorganisms

African elephants are megaherbivores consuming very large amounts of woody vegetation and fewer grasses characteristic of the savannah biome [97, 98]. Their woody diet contains about 17–30% of lignin [99]. Elephants ingest huge amounts of poor-quality forage due to physiological disadvantages such as lower gut surface area and short intestinal tract, which lead to poor extraction capacity of nutrients [5]. Literature suggests that the gut microbiome associated with an animals’ gut is mostly influenced by their diet and ecological niches [26, 100, 101]. Microorganisms inhabiting the gastrointestinal tract are known to be unique to their symbiotic hosts [3]. It follows therefore that elephants inhabiting other geographical regions, for example, Asian elephants, may harbour different ligninolytic microorganisms [102]. Interestingly, a comparative study on the digestion and intestinal tracts of Asian and African elephants suggests that the latter have shorter intestines, which therefore suggests a poor digestion coefficient than the former [103]. The potential to harbour robust ligninolytic microorganisms in African elephant guts to ensure the efficient breakdown of recalcitrant lignin associated with woody vegetation and a faster gut movement is more pronounced to facilitate efficient uptake of nutrients [5]. It is noteworthy that although ligninolytic bacteria found in ruminants and horses are like those found in elephants’ guts, there are unique microorganisms found in elephants important to increasing digestive efficiency of diets of lower quality [104]. While lignin is difficult to depolymerise without oxygen, the anaerobic conditions of the elephant GIT may prove to be advantageous as anaerobic conditions are necessary for industrial fermentations [48] and lignin depolymerisation under aerobic conditions is more challenging as it cannot be degraded by low redox potential oxidoreductases due to the three-dimensional complex that acts as a barrier [48, 105].

Although the type of food ingested and the ecosystem of gastrointestinal tracts, characterised by several chemicals, environmental, and physical conditions functionally select for the best ligninolytic microorganisms, the environment inhabited by the elephants also influences the robustness of the microorganisms as a desirable characteristic of ligninolytic microorganisms. Botswana has arid to semi-arid savannah biomes with unpredictable rainfall patterns that are characterised by seasonally higher desert-like temperatures as well as hypersaline environments [97, 106]. Such environments are characteristic of extreme environments with the potential to select for and harbour extremophiles. The specific differences in types of lignin wastes generated from different industrial sectors reduce the potential of finding a single best host for lignin valorisation. Desirable ligninolytic microorganisms must have extremophilic microorganisms with desirable stress tolerance for industrial conditions [21]. For example, high tolerance for inhibitors such as sulphate [28] associated with kraft lignin as the most abundant lignin waste is a highly desirable trait of interest. In addition, over a third of Africa’s elephant population [106] inhabit protected game reserves such as the Chobe National Park, Central Kalahari Game Reserve (CKGR) and the Makgadikgadi salt pans game reserves which are characterised as pristine environments completely isolated from anthropogenic activities and pollution. Anthropogenic activities and pollution perturb microbial species richness and distribution [100]. Such environments suggest that there is a potential to find novel ligninolytic microorganisms with potentially undescribed metabolic pathways. Furthermore, the yearly migratory behaviour of Botswana elephants from the salty regions of the Makgadikgadi Pans to the warm areas of Chobe and the Okavango delta as well as to neighbouring Angola, Zambia, and Zimbabwe Trans frontier Park (characteristic of different temperatures, climates, vegetation, and water access) suggest that their microflora should be flexible to the changing environments. The ability of ligninolytic microorganisms to adapt to different environments is a desirable attribute of efficient industrial strains [88].

## Extremophilic ligninolytic microorganisms: a key trait sought in production strains

A recalcitrant biopolymer like lignin requires microorganisms with robust traits. Extremophilic microorganisms with the ability to survive in harsh environments could have such unique credentials to remain metabolically efficient in stressful industrial conditions [107]. Production strains are subjected to stressful exposure to nutrient starvation, pH, temperature, inhibitory and toxic chemicals, osmotic stress, and others [88]. Literature suggests that extremophilic microorganisms have phenotypic plasticity that allow them to cope in such heterogeneous and stressful environments, adapt and thrive even in sublethal conditions [108]. Unexplored metabolic pathways characteristic of elephant guts microbiota could be responsible to produce robust enzymes with some credentials worth exploring as a fundamental background for the future of sustainable biorefineries [123]. Examples of innate survival mechanisms characteristic of extremophiles which could withstand very harsh industrial conditions include the ability to tolerate high temperatures, ability to remain metabolically active in the presence of process inhibitors (phenolic compounds, inorganic ions, furan derivatives, organic acids, and others) linked to lignin pre-treatment processes [28]. Production strains must therefore be able to withstand the ever changing and harsh industrial conditions. Production strains selected for the valorisation of lignin need to be highly stable [88] because varying industrial conditions could lead to diverse yields of desired low molecular weight compounds characteristic ligninolytic processes [33]. Examples of traits of importance for lignin-valorising microorganisms are shown in Table 2. The table generally suggests the diverse extremophilic traits desirable in the biorefinery processes [109, 110]. Lignin-enriched media is a common method to screen for potential ligninolytic microorganisms, lignin and xylan-amended enrichment of Eastern Mediterranean seawater provided for a suitable selective media for ligninolytic and extremophilic microorganisms [111].

**Table 2:** Extremophilic traits of ligninolytic bacteria

Microorganism	Extremophilic trait and its importance in parentheses	Ligninolytic activity	Method of isolation	References
*Acetoanaerobium sp.*	Anaerobic (low-cost production in absence of oxygen)	Oxidation of lignin	Kraft lignin enrichment medium	[43]
*Clostridium sp.*	Thermophilic (higher process temperatures minimising cooling costs and biological contamination, increases reaction rates, reduces product viscosity)	*O*-demethylation of aromatic compounds	Lignin and lignin model compound (guaiacol)-enriched medium	[88, 109, 112, 113]
*Geobacillus thermodenitrificans Y7*			CMC fermentation medium	[114, 115]
*Thermobifida fusca*		Oxidation of lignin	Alkaline lignin assay	[42, 116], 117]
*Thermus thermophilus*			Kraft lignin-enriched medium	[118]
*Arthrobacter sp.*	Psychrophilic (saves energy and reduces production costs in low-temperature fermentation)	Oxidation of hydroxyl groups in lignin	Sodium lignin sulfonate-enriched medium	[119]
		Demethylation of lignin Splitting of carbon–oxygen bonds in lignin		
*Halomonas sp.* *Aquisalibacillus elongatus*	Halophilic and halotolerant (remain stable in organic solvents)	Ring cleavage of lignin by 3,4-dioxynase and additional enzymes	LB enriched with synthetic lignin model compounds	[88, 120] [121]
*Burkholderia* sp.	Acidophilic (may have secondary transporters, high membrane impermeability, membrane potential reversal allowing for broad applications)	Meta-cleavage of catecholic intermediates in lignin	Alkaline lignin-enriched medium	[32, 122]
*Bacillus ligninphilus L1*	Alkaliphilic (used in depolymerisation of kraft lignin without adjusting pH (more economically feasible)	Lignin aryl-ring degradation (β-ketoadipate pathway)	Lignin-enriched medium medium	[25, 88]
*Gammaproteobacteria*	Oligotrophic (tolerate high ionic strength conditions)	Lignin aerobic depolymerisation via phenylacetyl-CoA thioesters	Lignin–xylan amended enriched medium media	[111]

## Ligninolytic microorganisms from other animal guts other than elephants

The gut of herbivores is generally considered a putative reservoir for ligninolytic microorganisms [80, 123, 124]. Microflora from other herbivores’ guts such as monogastric herbivores (such as rhinoceroses, rabbits, and horses) and ruminants (cattle, giraffes, and deer) as well as their faecal excreta, have also been documented as attractive sources [125, 126, 127, 128]. Their lignin-rich diet qualifies them as a source of ligninolytic microorganisms. Ligninolytic microorganisms from termite guts have also been extensively studied [129, 130]. Termite guts harbour lignin-degrading bacteria belonging to varied genera such as *Streptomyces, Pseudomonas, Bacillus and Sporothrix* [129]. An extensive review of dung beetles by Nwaefuna et al., [131], also suggested that exploration of dung (excreta) of non-herbivores insects is becoming attractive in the search for ligninolytic microorganisms. Identification of ligninolytic consortia present in different animal guts is increasingly becoming easier using state-of-the-art high-throughput techniques [13, 132, 133].

## Current methods and advances in the discovery of ligninolytic microorganisms from herbivore guts and dung

### Isolation, screening, and detection of metabolites methods

In the quest to isolate ligninolytic microorganisms, one of the key factors to first consider is the sample source, which is highly probable to be rich in organisms with a ligninolytic phenotype such as lignin-rich environments [27]. The gastrointestinal tract and dung of herbivores and grazers are very attractive niches that have yielded many ligninolytic microorganisms [134]. There are several methods for isolating microorganisms with elevated ligninolytic activity. Use of enrichment media either in liquid or on solid media to screen for microorganisms that can utilise lignin as a sole carbon source and selecting against those that cannot utilise it [41, 42]. These methods can be done under anaerobic conditions to mimic the gastrointestinal tract environment of the elephant to isolate organisms harbouring major enzymes that function under anaerobic conditions such as phenyl phosphate synthase [85], glutathione and Dyp-type peroxidases identified from the anaerobic *Klebsiella* sp. strain BRL6-2 [135]. Glutathione S-transferase genes for lignin degradation were also identified from the facultative anaerobe *Enterobacter lignolyticus* SCF1 [70]. Isolation of ligninolytic microorganisms under aerobic conditions is well documented but does not represent the anoxic environment in the GIT but allows the isolation of microbes harbouring oxidative enzymes such as peroxidases, laccases, polyphenol oxidases, phenol oxidases [85, 136], possibly from oxygen-exposed excreta of elephants. Although quite difficult to depolymerise lignin without oxygen, anaerobic conditions are economically attractive to produce valuable products by using a less expensive anaerobic fermentation process as compared to costly aerobic fermentation processes [48]. Examples of bacteria able to depolymerise lignin under anaerobic conditions include *Rhodococcus jostii* [64], *Tolumonas lignolytica* [51], *Enterobacter lignolyticus* [137]. If solid media is used, colonies that emerge will suggest their ability to utilise lignin sources. A dye decolourisation method involving the supplementation of the media with specific dyes and observing colour changes can also be incorporated [65, 138, 139]. Dye decolourisation can also be used to indicate potential enzymes responsible for lignin depolymerisation. For example, lignin peroxidase activity may be observed using methylene blue decolourisation [140]. In most cases colony growth can be qualitative but, in some cases, advanced imaging and software can be used to quantitatively determine the size of the colonies. The size of the colony can be correlated to the utilisation ability. A quantitative method entailing the growth of pure isolates in lignin-enriched liquid media (lignin as the sole carbon source), and then the determination of the rate of growth and rate of disappearance of lignin is more accurate. Colorimetric assays [90, 117, 141, 142] and fluorescence assays provide a unique approach to tracking the change in the two attributes when lignin is broken down [117, 143] while other methods such as lignin enrichment media show the potential of a lignin-degrading microorganism through observation of growth on a lignin-enriched media [82]. Strains that have gone through primary screening methods further go through secondary screening where more detailed screening is done. Secondary screening answers questions such as yield potential and subsequent industrial applicability of isolated strain [144]. The use of chromatographic and spectroscopic methods such as GC–MS, HPLC and UPLC are typical methods that have been widely used to determine and quantify products of lignin degradation. Recently, *Bacillus ligniniphilus* L1 was reported to have produced 15 unrelated aromatic compounds with vanillic acid produced in the highest proportion of 42% using these methods, which could have not been the case with conventional methods [25]. Low molecular weight compounds such as ferulic acid, gallic acid, trimethoxy benzaldehyde have been identified from lignin depolymerisation using GC–MS [145].

## Molecular confirmation of ligninolytic activity and identification of ligninolytic isolates

Whilst a microorganism can be primarily screened to have ligninolytic potential, further confirmation can be done through molecular analyses of the genes encoding ligninolytic activity. The advent of next-generation sequencing has become a mainstay allowing multiple identifications of such genes. Genes encoding lignin peroxidases and manganese peroxidases have been reported using this technological advancement [146]. Whole-genome sequencing and associated bioinformatics tools are now being used to predict the putative genes for the catabolism of lignin and the derived aromatic derived compounds [59] [147, 148]. For example, *Lac51* gene identified from a metagenomic analysis of panda faeces was found to oxidise a variety of lignin-related compounds such as ferulic acid, guaiacol under conditions mimicking the panda GIT [149]. Polyphenol oxidase capable of degrading lignin-related compounds was also identified from a metagenomic expression library of bovine rumen [150]. A dye decolourising peroxidase gene and the genome-based metabolic reconstruction and other lignin modifying enzymes (LMEs) and lignin-degrading auxiliary enzymes (LDAs) have been documented [151]. In addition, several gene clusters responsible for aromatic depolymerisation [111], classes mediating lignin transformations have been identified [152], for example, ring hydroxylating oxygenase [153], *bph*A_1_A_2_BCD [154], class II peroxidase genes [155].

Identification of isolates to species level using strictly conserved sequences of the ribosomal RNA genes in both prokaryotes and eukaryotes is a common method utilised in modern biotechnology. If a full taxonomic resolution is required, then a full gene, multiple loci and whole-genome analyses have become attractive to resolve the molecular differences. The advent of metagenomics technologies is slowly becoming useful, for example, studies using this technique on the *Anoplophora glabripennis* dung beetle microbiota have also reported on the presence of genes encoding enzymes with lignin depolymerisation capability such as laccases, dye decolourising peroxidases, β-etherases [156].

## Strategies to improve ligninolytic activity of microorganisms from elephant guts

The challenge of finding robust ligninolytic strains to efficiently utilise recalcitrant lignin remains a major obstacle in lignin wastes valorisation. Naturally, microorganisms may harbour ligninolytic metabolic pathways albeit with a poor ability to depolymerise lignin for profitable commercial scales and depolymerisation in the presence of pre-treatment products creating adverse habitat-irrelevant industrial conditions. It is therefore imperative to improve the ligninolytic activity as well as the productivity of potential industrial strains. Rational approaches to developing the best performing production strains have become attractive in developing production strains with improved industrially suitable traits have been extensively reviewed [157]. Chemical and radiation mutagenesis, hybridisation, directed evolution, synthetic biology, and targeted metabolic engineering are common. Hybridisation has been used to create novel strains in other fields [157], but none have been described in lignin valorisation processes. However, hybridisation success frequency happens at a low rate, and it may be difficult to identify hybrids [158, 159]. With directed evolution, there are many positives such as no requirements for prior genetic knowledge of the strain being developed and relative ease of carrying out the procedures, among others [160]. Although other negative outcomes of this method such as genetic hitchhiking, clonal interference, physiological heterogeneity among evolved populations [161, 162, 163], it can be used to optimise many industrial strains [157]. Synthetic biology is another powerful route that can be used although designing and optimising robust strains for industrial operations is a challenge [164]. Metabolic engineering manipulates various pathways of desired secondary metabolites to improve their yield, however, the biosynthetic pathways to produce the desired products using wild and novel isolates are often unknown [165].

## Conclusion and future directions

The use of plant biomass as an inexpensive and abundant renewable source of industrial substrates and energy is attractive for a sustainable global economy. However, as discussed in the review, the accumulation of bulky recalcitrant lignin waste from the pulp and paper, biofuel and agro-industries is a major drawback to the feasibility of the technologies. The review presented the biological valorisation of recalcitrant lignin in an integrated biorefinery concept as an attractive strategy for increased sustainability of biorefineries. The review presented the potential in bioprospecting for elephant-associated lignin-degrading microorganisms as an attractive strategy for the valorisation of lignin wastes towards viable and cost-effective biorefineries. The uniqueness of the abundant elephants, the varied climate conditions and the pristine biome structure could have shaped life-history strategies of ligninolytic microorganisms. Novel microorganisms and undiscovered taxa may offer unique ligninolytic capabilities to manage lignin waste streams and produce specialty chemicals for a circular bioeconomy.

## Data Availability

Not applicable.
